# *Mycobacterium tuberculosis* DosR Regulon Gene *Rv0079* Encodes a Putative, ‘Dormancy Associated Translation Inhibitor (DATIN)’

**DOI:** 10.1371/journal.pone.0038709

**Published:** 2012-06-13

**Authors:** Ashutosh Kumar, Mohammad Majid, Ralph Kunisch, Pittu Sandhya Rani, Insaf A. Qureshi, Astrid Lewin, Seyed E. Hasnain, Niyaz Ahmed

**Affiliations:** 1 Pathogen Biology Laboratory, Department of Biotechnology, School of Life Sciences, University of Hyderabad, Hyderabad, India; 2 Robert Koch Institute, Berlin, Germany; 3 Department of Biotechnology, School of Life Sciences, University of Hyderabad, Hyderabad, India; 4 School of Biological Sciences, Indian Institute of Technology, New Delhi, India; 5 Institute of Life Sciences, University of Hyderabad Campus, Hyderabad, India; 6 King Saud University, Riyadh, Saudi Arabia; 7 Institute of Biological Sciences, University of Malaya, Kuala Lumpur, Malaysia; Institute of Microbial Technology, India

## Abstract

*Mycobacterium tuberculosis* is a major human pathogen that has evolved survival mechanisms to persist in an immune-competent host under a dormant condition. The regulation of *M. tuberculosis* metabolism during latent infection is not clearly known. The dormancy survival regulon (DosR regulon) is chiefly responsible for encoding dormancy related functions of *M. tuberculosis*. We describe functional characterization of an important gene of DosR regulon, *Rv0079*, which appears to be involved in the regulation of translation through the interaction of its product with bacterial ribosomal subunits. The protein encoded by *Rv0079*, possibly, has an inhibitory role with respect to protein synthesis, as revealed by our experiments. We performed computational modelling and docking simulation studies involving the protein encoded by *Rv0079* followed by *in vitro* translation and growth curve analysis experiments, involving recombinant *E. coli* and Bacille Calmette Guérin (BCG) strains that overexpressed *Rv0079*. Our observations concerning the interaction of the protein with the ribosomes are supportive of its role in regulation/inhibition of translation. We propose that the protein encoded by locus *Rv0079* is a ‘dormancy associated translation inhibitor’ or DATIN.

## Introduction

Tuberculosis (TB) is a chronic infectious disease caused by *Mycobacterium tuberculosis* which is linked to high morbidity and mortality worldwide. *M. tuberculosis* can exist in active form or can remain alive in a dormant state in lungs after forming granuloma where it can prolong its persistence without replication [1]. The tubercle bacilli in the dormant or latent state may not be affected by antibiotics or the host immune system due to their bare minimum growth [2], [3]. Several studies have indicated that, under latency, the granuloma offers a niche with increased concentration of nitric oxide, low oxygen and absence of nutrients. To survive under such unfavourable conditions [2]–[6], *M. tuberculosis* might have evolved mechanism(s) whereby it decreases the rate of protein synthesis to conserve its cellular resources. Such mechanisms have not been clearly deciphered.

*M. tuberculosis* genome encodes a regulon of 48 constituent genes called the dormancy survival regulon (DosR regulon) [6]. Inhibition of aerobic respiration causes up-regulation of the transcription factor, DosR, pointing out that the control of the regulon is related to physiology of respiration in *M. tuberculosis* [6]. Although many of the constituent genes of DosR regulon encode hypothetical proteins, growing knowledge of the conditions under which these genes are likely up-regulated could lead to their role in adaptation of *M. tuberculosis* to the host environment [6]. The humoral immune response for DosR regulon encoded antigens is stronger in latently infected individuals when compared to individuals with active infection [7] suggesting that DosR regulon genes are more likely expressed during latency. Many proteins encoded by the genes of this regulon are thought to be helpful to obtain energy from alternative sources of carbon such as glyoxylate metabolism, nitrate reduction and fatty acid metabolism [6], [8]. Several of the members of DosR regulon which might be significant in understanding dormancy regulation could not be functionally characterized as yet. *Rv0079* is the first member, by order [6], in the DosR regulon that has not been functionally characterized, although it has been computationally shown to encode a predicted translation factor [9].

Here we describe functional characterization of the protein encoded by *Rv0079*. The gene was found to be conserved in all the clinical isolates analysed by us, and also in almost all the publicly available *M. tuberculosis* genomes. *In silico* structural analyses predicted the ability of *Rv0079* encoded protein (henceforth called DATIN) to dock at the 30S ribosomal subunit. Furthermore, we identified that DATIN inhibits *in vitro* protein synthesis from mRNA templates. Its own synthesis was arrested when overexpressed in *E. coli* BL21 (DE3) cells, and it decreased the growth rate of *E. coli* BL21 (DE3) and BCG upon over-expression.

## Materials and Methods

### Ethics Statement

This work did not involve any animal experiments or the use of patient material or human biological samples. All recombinant DNA work reported in the manuscript was approved by the institutional biosafety committee of the School of Life Sciences, University of Hyderabad.

### Distribution of the Locus *Rv0079* in *M. tuberculosis*

Distribution of *Rv0079* gene was analyzed in 10 randomly taken clinical isolates of different geographical origins. PCR amplification of *Rv0079* was carried out using 100 ng of genomic DNA, 10 pMol of each primers (see sequences in the next sections), forward and reverse (designed to target the locus), 200 µMol of each deoxynucleoside triphosphate, and 1 unit of *Pfu* DNA polymerase (Fermentas Inc., USA) in a standard PCR buffer supplied by the manufacturer. Amplification was performed in a Master Cycler gradient PCR machine (Eppendorf, Germany) under the following conditions: an initial denaturation at 95°C for 10 min was followed by 30 cycles of 94°C for 30 sec, 60°C for 45 sec, 72°C for 1 min 30 sec, and a final extension at 72°C for 15 min. PCR products were separated by electrophoresis in 1% agarose gel and visualized under UV light.

### Computational Analysis and Structure Prediction

**Figure 1:**
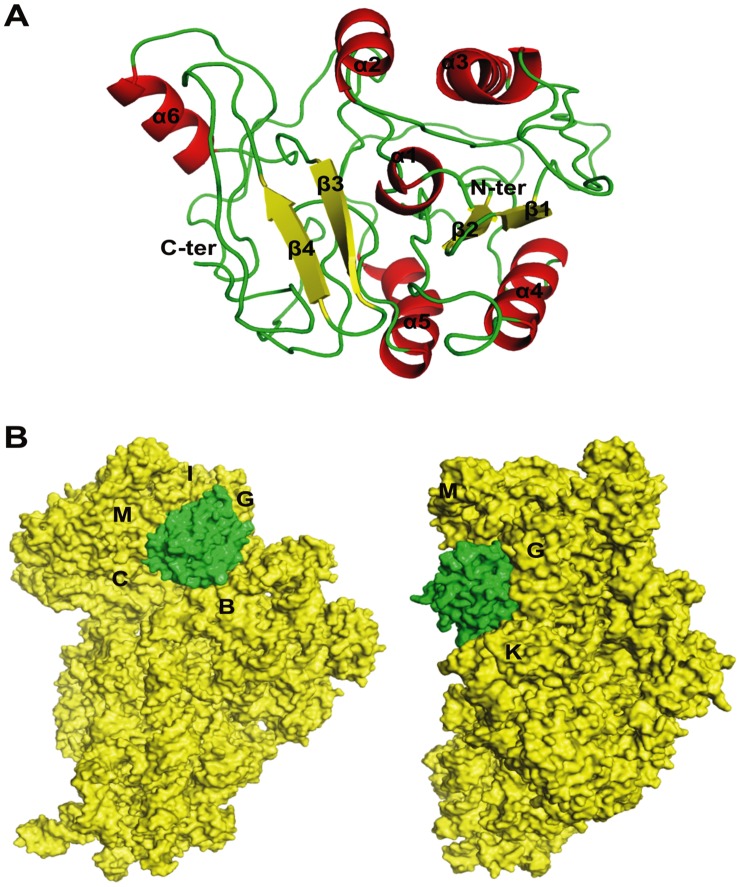
Predicted 3D-structure of DATIN and its docking simulation with 30S ribosomal subunit. A. Predicted 3D-structure of DATIN. The protein secondary structure elements were labelled and colored (helices and sheets displayed in red and yellow, respectively). B. Docking study of DATIN with 30S ribosomal proteins using PatchDock and FireDock. The protein chains of 30S ribosome and DATIN presented as electrostatic surface and colored in yellow and green, respectively.

*In silico* sequence analysis and computational modelling of DATIN (Figure 1) was performed using different tools such as DAS [10], ConPred II [11], TOPCONS [12], and PSORTb v3.0 [13] to specifically analyze for the hydropathy index, membrane topology and surface probability. Amino acid sequences of DATIN were submitted for 3-dimensional structure predictions at I-TASSER server [14]. Structure was selected on the basis of RMSD values and agreement with Ramachandran Plot, available with PDBsum program [15]. Molecular visualization and general analysis were carried out using the program PyMOL [16]. *In silico* docking experiments were performed using PatchDock [17] and then further refined and ranked with FireDock [18]. Crystal structure of 30S ribosome (PDB ID: 2AVY) consists of RNA molecule and several protein molecules, and therefore only the protein molecules (‘receptor’) were taken for docking study with DATIN (‘ligand’) under default complex-type settings.

### Molecular Cloning and Engineering of Recombinant Bacterial Strains

The construct for recombinant protein expression was generated by amplifying the *Rv0079* gene using forward GCC**CTCGAG**GTGGAACCGAAAC (*Xho*I) and reverse CGA**AAGCTT**GCTCATGCCAGAC (*Hin*dIII) primers. Amplified product and pRSET-A (Invitrogen, CA, USA) vector were double digested by *Hin*dIII and *Xho*I enzymes and kept for overnight ligation at 16°C. The clone was confirmed by releasing the insert after restriction digestion and sequencing with T7 primer. The construct of *Rv0079* was then transformed into *E.coli* BL-21 (DE3). The gene locus *Mb0082* of *M. bovis* BCG displays 100% sequence identity with the gene *Rv0079* of *M. tuberculosis*. An *Rv0079* or *Mb0082*-overexpressing recombinant BCG [BCG (pMV261+*Rv0079* or *Mb0082*)] was generated by amplifying the coding sequence (using *M. bovis* BCG DNA as template) with the help of primers [Rv0079_FP_*Hin*dIII (CC**AAGCTT**GTGGAACCGAAACGCAGTCG) & Rv0079_RP_*Hpa*I (CC **GTTAAC** TCATGCCAGACCGTCGGC)] and inserting the PCR product downstream of the *hsp*60 promoter in the *Hin*dIII/*Hpa*I sites of the vector pMV261 [19]. After confirmation of the absence of any mismatches by sequencing, the recombinant plasmid as well as empty vector, pMV261 were introduced by electroporation into BCG as described before [20].

### Expression and Purification of His- Tagged Recombinant Protein

DATIN was produced by transforming *E. coli* BL21 (DE3) cells with the construct generated for recombinant protein expression and the transformed colonies were picked up using ampicillin selection. Recombinant *E. coli* BL21 (DE3) cells were grown in Luria-Bertani broth (containing 100 µg/ml ampicillin) up to a cell-density (OD_600_) of 0.4–0.6 and then induced by IPTG (Sigma, USA) followed by incubation at 37°C for 4 hr. The culture was centrifuged at 6000 rpm and the cell-pellet was lysed in 20 mM Tris-HCl and 200 mM NaCl of pH 8.0 (lysis buffer) by sonication. The resultant lysate was centrifuged at 12000 rpm for 45 min at 4°C and its supernatant was loaded on cobalt based resin (TALON® Metal Affinity Resins, Clontech) to purify His-tagged DATIN. The column was washed extensively with washing buffer (lysis buffer with 10 mM imidazole; pH 8.0) and the over-expressed His-tagged protein was eluted using elution buffer (lysis buffer with 250 mM imidazole). Further, size exclusion chromatography was performed using Superose12 10/300 GL column (GE Healthcare Ltd.) in a buffer containing 20 mM Tris-Cl and 300 mM NaCl (pH 8.0) and the protein profile was compared with protein molecular size standards. The recombinant protein was quantified using Bradford’s reagent [21] and the purified protein was stored at −20°C until further use.

### *In Vivo* Translation Inhibition Assay

The *Rv0079* and *HP0023* (encoding isocitrate dehydrogenase of *Helicobacter pylori*) genes were cloned separately into pRSET-A vector for high-level protein expression of cloned genes in *E. coli*. Later, the constructs were used to transform *E. coli* BL21 (DE3) cells and the recombinant colonies were selected using ampicillin. The recombinant *E. coli* grown up to a cell-density (OD 600) of 0.4–0.6 were induced with 0.25 mM IPTG (Sigma, USA) and the cell pellet was collected at every 2 hr interval for 24 hr. The cell pellet was lysed in 20 mM Tris-HCl and 200 mM NaCl (lysis buffer, pH 8.0) using sonication. The resultant lysate was centrifuged at 12,000 rpm for 45 min at 4°C. Equal amount of protein was loaded on 12.5% SDS-PAGE gel and western blotting was performed to detect the expression level of the desired protein(s). The probing antibodies comprised of anti-His mouse IgG (primary) and HRP conjugated goat anti mouse IgG (secondary) (Santa Cruz Biotechnology Inc, USA). The signal was detected using VersaDoc imaging system (Bio-Rad).

### *In Vitro* Translation Inhibition Assay

To identify the role of DATIN in protein synthesis inhibition, coupled *in vitro* transcription/translation of circular DNA templates was carried out by pBEST*luc*™ containing the firefly luciferase gene and *E. coli* S30 Extract System (Promega Corporation, USA). Three sets of coupled *in vitro* transcription/translation were carried out. The reaction mixtures contained 2 µl of pBEST*luc*™ DNA (1 µg/µl), 5 µl of amino acid mixture minus methionine, 20 µl of S30 premix without amino acids, 15 µl of S30 extract circular and 1 µl of (35S) methionine (1,200 Ci/mmol at 15 m Ci/ml). Two µg of DATIN and 2 µg of BSA were added in the second and third reaction mixtures, respectively. In the first set of reaction mixture, Milli-Q water was added instead of DATIN or BSA. The reaction mixtures were incubated at 37°C for 90 min, then the tubes containing reaction mixtures were placed in ice to stop the reaction. Aliquots (5 µl) of each reaction were loaded onto 12.5% SDS-PAGE gel to analyze the translation product. Electrophoresis was carried out at a constant voltage of 50 volt in stacking gel and 100 volt in separating gel. Following electrophoresis, gels were dried, exposed overnight to a phosphorscreen (Amersham Biosciences), and scanned with a Typhoon 9410 variable mode imager.

### Growth Experiments in Broth Culture

To see the effect of DATIN on the growth of the bacteria, comparison of the growth rates of the strain overexpressing *Rv0079* [*E. coli* BL21(pRSETA+*Rv0079*)] and the strain which does not express *Rv0079* [*E. coli* BL21(pRSETA)] was carried out. The recombinant *E. coli* BL21(pRSETA+*Rv0079*) and *E. coli* BL21(pRSETA) were generated by transforming the *E. coli* BL21(DE3) cells with a recombinant construct of pRSETA vector containing *Rv0079* and, an empty pRSETA, respectively, and the recombinant colonies were picked up using ampicillin selection. Both the strains were inoculated in Luria Bertani broth containing 100 µg/ml ampicillin with an initial optical density (OD_600_) of 0.04 and ODs of the cultures were measured at hourly intervals after inoculation. IPTG (Sigma, USA) was added in both the cultures when recombinant *E. coli* were grown up to a cell-density (OD_600_) of 0.4–0.6.

Further, to confirm the role of DATIN in a mycobacterial background, an *Rv0079*-overexpressing recombinant BCG [BCG(pMV261+*Rv0079*)] and BCG containing empty vector BCG(pMV261) were inoculated in Middlebrook 7H9 media supplemented with oleic albumin dextrose catalase and containing 0.05% Tween 80 and 25 µg/ml kanamycin. OD values of the cultures were measured at 4 days intervals after inoculation.

### Toxicity Assay

To assess the toxic effect, if any, of DATIN on *E.coli*, a single colony of *E. coli* BL21(pRSETA+*Rv0079*) was inoculated in Luria Bertani broth containing 100 µg/ml ampicillin and kept at 37°C in shaking incubator for overnight. Diluted overnight grown culture was plated on two sets of LB–ampicillin agar plates, one set containing 0.25 mM IPTG and the second one without this inducer.

### *(In Vivo*) DATIN-ribosome Interaction

The ribosomes were isolated from *E. coli* BL21(transformed with pRSETA+*Rv0079*) and the *E. coli* BL21(pRSETA+vector alone). To isolate the ribosomes, sonication of bacterial cells was performed and the cells were resuspended in a buffer containing 20 mM Hepes-KOH of pH 7.8, 10 mM MgCl_2_, 60 mM NH_4_Cl, 4 mM 2-mercaptoethanol, 1 µg/ml lysozyme and 0.2 mM phenylmethylsulfonyl fluoride. Lysates were clarified by centrifugation at 10000 rpm for 30 minutes; supernatant was then centrifuged at 20000 rpm for 1 hr followed by centrifugation again at 40000 rpm for 4 hr. The pellet thus obtained was dissolved in the same buffer (as above) and centrifugation was done for 2 hr at 40000 rpm to achieve pure ribosome fraction. All the steps of ribosome isolation were performed at 4°C and the purified ribosomes were kept at −80°C for further use. Equal amounts of purified ribosomes were loaded on a 15% SDS-PAGE gel and western blotting was done by probing with antibodies against 6X His tag to detect the presence of DATIN bound with the ribosome(s).

## Results

### Characterization of *Rv0079* and the Recombinant Protein (DATIN)

*Rv0079* was found intact in all the 10 clinical isolates that we tested and in the sequenced genomes of *M. tuberculosis* F11, *M. tuberculosis* W-148, *M. tuberculosis* KZN 4207 (DS), *M. tuberculosis* KZN 605 (XDR), *M. tuberculosis* 98-R604 INH-RIF-EM, *M. tuberculosis* Haarlem, *M. tuberculosis* C, *M. tuberculosis* KZN 1435 (MDR), *M. tuberculosis* H37Rv, *M. tuberculosis* H37Ra and *M. bovis* BCG. The gene was found intact and normally located in both the ancestral and modern lineages of *M. tuberculosis*. This tight conservation of the locus in all the strains representing different geographical origins and clinical backgrounds points to the functional importance of DATIN for mycobacterial lifestyle.

In the toxicity assay, the average number of colonies of *E. coli* BL21 (pRSETA+*Rv0079*) grown on the LB –ampicillin agar plates were 40.33 and on LB–ampicillin agar plates containing IPTG were 39. Presence of an almost equal number of colonies on both types of plates nullifies the probability of any toxicity [22] of DATIN towards *E. coli* BL21 cells. This confirms that decrease in the growth rate of *E. coli* expressing *Rv0079,* that we recorded (see later) in broth cultures, was not due to any toxic effect of recombinant heterologous protein, but may be due to its interaction with or impact on the ribosomes (Figure 1) (that possibly slows down translation rate). To determine/confirm the putative biological function, *Rv0079* was over-expressed in *E. coli* and purified to homogeneity under native conditions as His-tagged protein. The elution profile of the recombinant protein confirmed monomeric nature of DATIN in solution. The purified protein on a 12.5% polyacrylamide gel showed a single band corresponding to ∼29.5 kDa after staining with Coomassie Brilliant Blue dye.

### DATIN Impacts Protein Synthesis and Bacterial Growth

**Figure 2:**
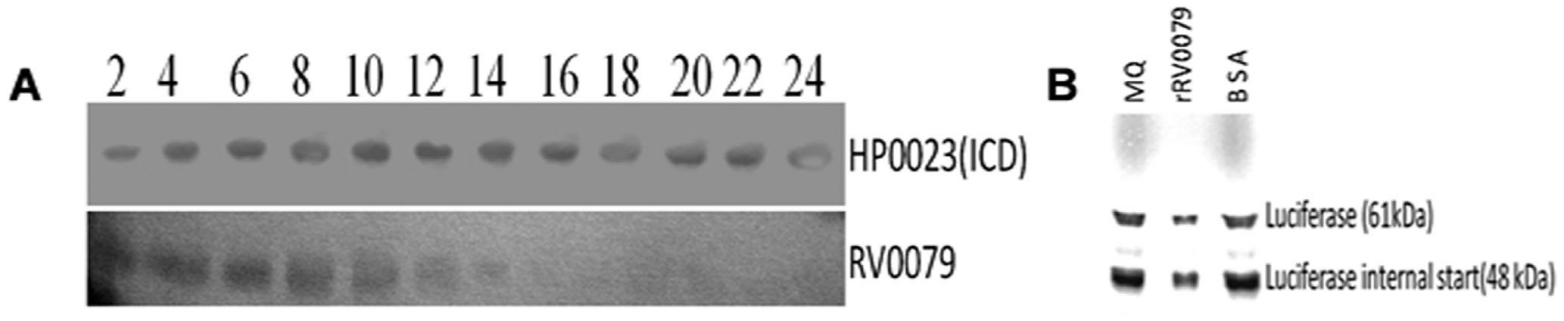
Protein synthesis inhibition by DATIN. A: Inhibition of protein synthesis *in vivo.* There was a decrease in DATIN production in recombinant *E. coli* over-expressing *Rv0079* gene after 10 hr of incubation. *E. coli* expressing *H. pylori* isocitrate dehydrogenase (*HP0023*) was taken as a control. B: Coupled *in vitro* transcription/translation of circular DNA templates using *E. coli* S30 Extract System for Circular DNA. Reaction mixtures contained Milli-Q water (lane 1), DATIN (lane 2) and BSA (lane 3) (an unrelated control). Full-length luciferase migrated at 61 kDa. An apparent internal translation start resulted in a second major gene product of 48 kDa.

Both DATIN and rHP0023 (an unrelated negative control) were produced in *E. coli* BL21 expression system separately. The western blot analysis of rHP0023 and DATIN indicated that synthesis of DATIN gradually decreased at durations from 10 hr to 24 hr, whereas, synthesis of rHP0023 was consistent even after 24 hr (Figure 2, panel A). This result suggests inhibitory nature of DATIN towards protein synthesis. *In vitro* protein synthesis was carried out by pBEST*luc*™ containing the firefly luciferase gene as a template. After completion of the reaction, it was observed that protein synthesis was diminished when 2 µg of DATIN was added to the reaction mixture. However, protein synthesis was not affected when similar reaction was performed using bovine serum albumin instead of DATIN (Figure 2, panel B).

**Figure 3:**
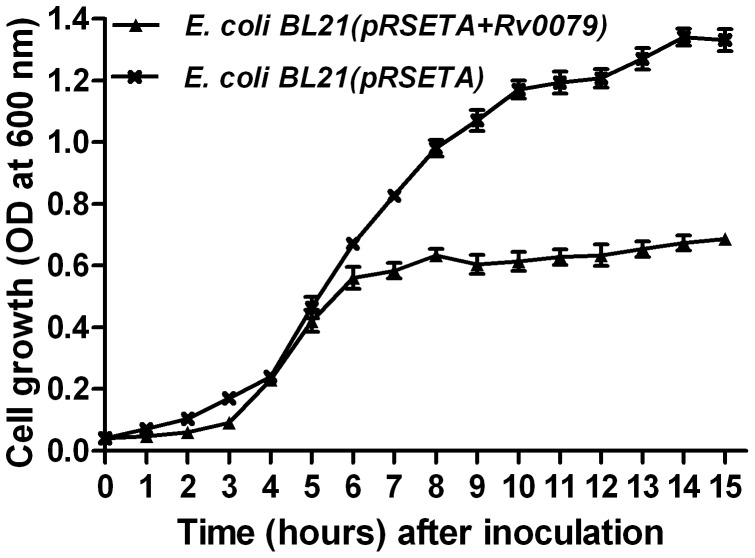
Growth (in broth) of recombinant *E. coli* expressing DATIN. Broth cultures from *E. coli* BL21(pRSETA+*Rv0079*) and *E. coli* BL21(pRSETA) were inoculated into Luria Bertani broth to give an initial OD (600 nm) of 0.04 and the cultures were incubated for 15 or 24 hr at 37°C under IPTG induction. The growth curves of the strains were generated by measurement of the OD at 600 nm. The values represent the mean of three independent shaking cultures with the standard deviation.

**Figure 4:**
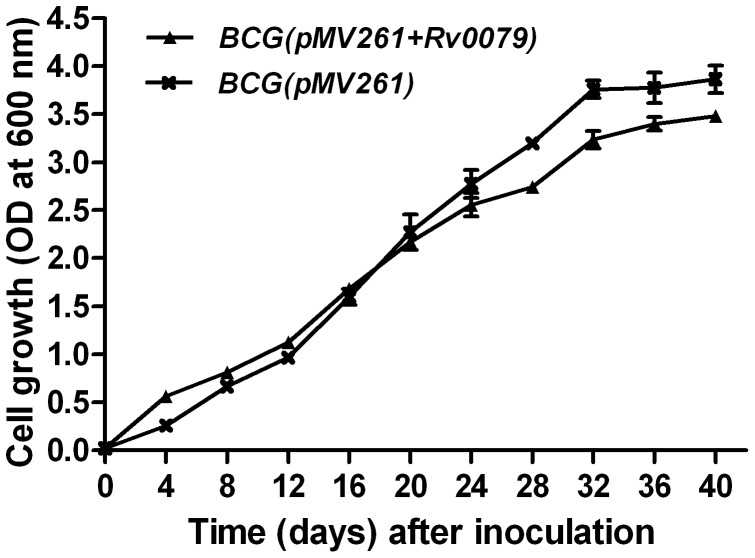
Growth (in broth) of recombinant *M. bovis* BCG over-expressing DATIN. Broth cultures from BCG (pMV261+*Rv0079*) and BCG (pMV261) were inoculated into Middlebrook 7H9 broth to give an initial OD (600 nm) of 0.02 and the cultures were incubated at 37°C as static cultures. The growth curves of the strains were generated by measurement of the OD at 600 nm. The values represent mean of the three independent ODs/static cultures with standard deviation.

The influence of DATIN on the growth rates of *E. coli* was determined by comparing growth curves of broth cultures from *E. coli* BL21(pRSETA+*Rv0079*) and *E. coli* BL21(pRSETA) (Figure 3). DATIN decreased the growth rate of the bacilli and also prevented them from achieving a higher cell density in the stationary growth phase. The OD values obtained with stationary phase cultures from *E. coli* BL21(pRSETA+*Rv0079*) were less than the OD values obtained with cultures from *E. coli* BL21(pRSETA) roughly by a factor of 2 (Figure 3). Also, the *E. coli* expressing *Rv0079* entered into the stationary phase much earlier (at 6 hr) than the *E. coli* not expressing *Rv0079* (at 10 hr) (Figure 3). This result was further confirmed by comparing growth curves obtained from the broth cultures of recombinant BCG [BCG(pMV261+*Rv0079*)] overexpressing *Rv0079* and the BCG containing empty vector [BCG(pMV261)]. As expected, BCG expressing *Rv0079* showed delayed growth rate compared to the control. The difference was statistically significant when the OD values after mid log phase were compared (Figure 4).

### DATIN Interacts with *E. coli* Ribosomes

The analysis for cytoplasmic or membrane localization using transmembrane prediction tools did not suggest any transmembrane segment in DATIN. Due to unavailability of crystallographic/solution structure of DATIN, the search for possible homologs was carried out using several programs. Sequence-based search methods (BLASTp) did not provide any significant hit but sequence search in PDB identified a template with 27% sequence identity. The template used in the analysis of DATIN was a bacterial ribosome binding protein (HI0257, PDB ID: 1IMU) from *Haemophilus influenzae* which showed similar fold at C-terminus in spite of low sequence identity between the two proteins. A total of 5 models were obtained using I-TASSER server and model number 1 was considered the best among them, based on energy considerations. The quality of the structure was assessed using Procheck and this displayed less than 2% discrepancy from the Ramachandran plot. Secondary structure analysis showed six alpha helices and four beta sheets in the modelled structure as shown in Figure 1(panel A).

In order to identify possible interacting domains of DATIN with 30S ribosomal subunit, PatchDock was employed for unbound protein-protein docking with 30S ribosome as a receptor and DATIN as a ligand. Approximately 100 predictions were generated using PatchDock and were submitted to FireDock to refine 10 best solutions on the basis of global energy. Possible binding interface residues were identified using 3D2GO binding site prediction server. Several of the lowest energy docking models emerging from this exercise placed DATIN on the side of the 30S ribosomal subunit. Among ten docked complexes, complex 1 was identified as the plausible one on the basis of minimum energy score and binding interface residues. A docking model of 30S ribosomal subunit with DATIN is shown in Figure 1(panel B). We found that helical regions 5 and 6 of DATIN have moved towards 30S ribosomal subunit and docked into a groove formed by the chains G, I and M (Figure 1). There was no indication of covalent bond involvement during docking, however, electrostatic forces were found to play major role in interaction.

**Figure 5:**
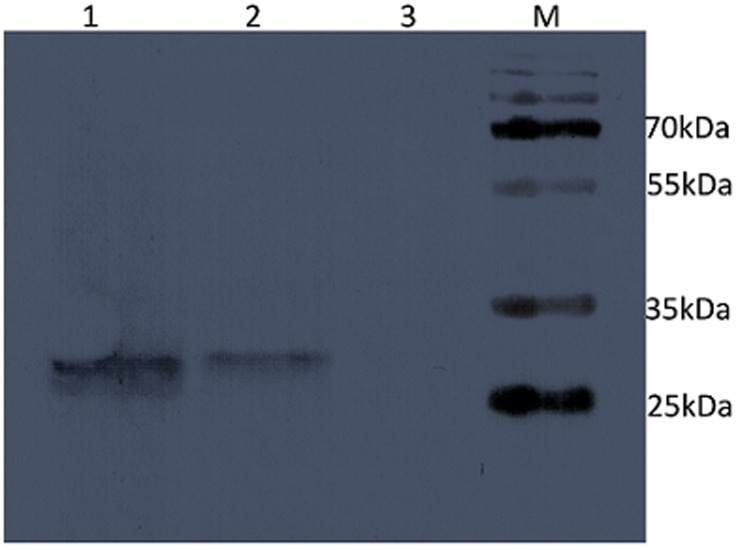
Binding of DATIN to *E. coli* ribosomes: Western blot detects purified ribosome preparation from *E. coli* cells (bound to overexpressed, His-tagged DATIN) probed with anti-His antibody (lane 2); *E. coli* cells containing empty vector (lane 3) served as negative control and purified DATIN (lane 1) was used as a positive control. Protein molecular weight marker (M) to indicate different size standards was run alongside.

Western blotting to detect any His-tagged DATIN fraction bound to purified *E. coli* ribosomes indicated that DATIN also migrated with the ribosomes. This means that DATIN remained bound to extracted ribosomes from *E. coli* overexpressing *Rv0079*. The negative control did not reveal any recombinant protein bound to ribosomal fractions (Figure 5).

## Discussion

As per the World Health Organisation (WHO) estimates, approximately 1.8 billion people around the world are infected with *M. tuberculosis* and a majority of the cases are clinically latent, waiting only to get activated upon immune-suppression [23]. Attempts to completely understand physiology and survival mechanisms of tubercle bacilli entailing latent TB have largely remained inconclusive. DosR regulon is one of the potential genomic co-ordinates in *M. tuberculosis* that are being aggressively studied to understand latency survival. The DosR regulon has been studied with respect to its regulation and expression but little is known about specific functions for DosR regulon proteins. Gene/protein sequence analysis and domain comparisons indicated that DATIN is likely involved in translation [9]. We, in this dossier, provide several pieces of evidence that support a role of DATIN in translation and ribosome regulation. We believe that these data are compelling, accurate and timely even though only a part of the work was conducted in a mycobacterial system.

Adaptation to the host niches during latency requires sensing of the stress factors and slowing of the metabolic activity in order to save energy. Sensing of the stress factors present in the granuloma is ensured by the sensor kinases which can autophosphorylate at a conserved histidine [24]. Transfer of the phosphate to the transcription factor DosR activates DosR regulon *via* transcription of 48 constituent genes of the regulon; these are up-regulated during oxygen stress [6], [24]. Slowing down of metabolic activity and saving of energy can be achieved by reducing the cell division rate and by storing macromolecular structures and preventing their degradation [25]. An effective reduction/inhibition of protein synthesis would likely arrest cell division and, the storage of ribosomes in an inactive state would save energy needed to synthesize ribosomal proteins and rRNA, which makes up major metabolic activity of bacteria. Storage of ribosomes during latency would also ensure their availability for protein synthesis if reactivation occurs. Both these aims might be achieved by the function of a factor that interacts with ribosomes to control translation.

Regulation of DosR regulon genes including DATIN is controlled by the cognate response regulator/transcription factor, DosR [26]. The latter binds to conserved consensus binding sequences (the dev boxes or dos boxes) present in the upstream promoter region of the DosR regulon genes, and thereby activates gene expression. Dev boxes have also been identified upstream of *Rv0079* [26]. Given this, one of the significant proteins that could potentially express under the conditions present in the granuloma would be DATIN.

*In silico* analysis of *Rv0079* encoded protein has previously shown that it might potentially act as a translation factor [9]; extending this thinking, we generated a further refined and resolved computational model with full protein sequence, and carried out a docking simulation followed by functional analysis of DATIN to elucidate its role in latency. We believe that the current model and, the envisaged interaction of DATIN with the ribosome(s) (Figure 1) actually strengthen the conceptualization of its role in translation and arrest of protein synthesis. Figure 5 reveals experimental evidence strongly suggestive of this possibility.

A role as translation factor or an inhibitor would require strong interaction with the translation machinery. The computational analysis of DATIN indicated that it has sequence homology to the ribosome binding protein of *Haemophilus influenzae*. Computational docking studies indicated that DATIN interacts with the domains of 30S ribosomal subunit. ProFunc [27], a computational tool to identify likely biochemical functions, based on a 3D protein structure, revealed that DATIN has similar folds like ribosome binding or ribosome associated proteins (for example, PDB codes 1IMU, 2YWQ, 3KA5 and 3LYV). In view of this, we carried out docking studies predicting DATIN’s interaction with 30S ribosomal subunit. This prediction points to a role for DATIN in stabilisation of 30S and 70S ribosomal subunits/ribosome and/or translation inhibition. To stabilize the translation machinery during stress/unfavourable conditions, certain dormancy related proteins might be expressed to possibly prevent the dissociation of the 50S and 30S. Upon attainment of favourable conditions the same ribosomal machinery can be used for protein synthesis. Further, there is also the possibility that this protein can work as a translation factor by interacting with 30S ribosomal subunit as has been hinted at by our growth experiments performed with *E. coli* and BCG and the *in vitro* translation assay (see later). Given this, we believe that the interaction apparently supports a more generalized mechanism of inhibition or arrest of translation. In order to investigate this role of DATIN, we tested its influence on protein synthesis *in vivo* and *in vitro*. We overexpressed *Rv0079* in *E. coli* and observed that the amount of DATIN produced therein declined after 10 hr of IPTG induction but the synthesis of an unrelated protein, rHP0023 was consistent even after 24 hr (Figure 2, panel A). This observation suggests that DATIN is potentially involved in translation. The function of DATIN in general translation inhibition was also supported by our *in vitro* protein synthesis experiments (Figure 2, panel B). While these *in vitro* translation data suggest that DATIN inhibits protein translation, it will be interesting to carry out similar translation inhibition studies in a mycobacterial background.

As mentioned above, the adaptation to the conditions in the granuloma also requires a reduction of the bacterial replication rate. To confirm this, we have grown recombinant *E. coli* and BCG in a series of experiments and it was observed that after mid-log phase the growth rates of the overexpressing strains were lower than those of the normal strains/controls (Figures 3 and 4). Furthermore, DATIN expression resulted in an early entry to the stationary phase of *E. coli* which in addition was characterized by a much lower cell density. Figure 3 shows in *E. coli* that induction of *Rv0079* expression by IPTG stops growth after 6 hr while this is not the case with a vector that does not contain *Rv0079*. This is an interesting and suggestive experiment that has become significantly stronger when at least one other protein (mycobacterial Hsp65) was expressed in the system to show that hyper-expression of any protein does not result in stasis (data not shown). Also, the observed growth inhibition was surely not caused by toxicity of the protein, as we have shown by a toxicity plate assay.

Recently, it has been reported that overexpression of hypoxia response regulator, DosR, leads to about 2 fold or greater transcription of 38 out of the 48 genes of DosR regulon, with a 10 fold up-regulation of the product of *Rv0079* [28]. However, it is not clear as to which gene/protein is responsible for the delayed growth or the maintenance of a non-replicating bacteriostasis; given our findings, it appears quite probable that DATIN could be potentially responsible for slowing the bacterial growth.

Collectively, these results lead to the thinking that DATIN might play a significant role in latent phase of *M. tuberculosis* infection by its ribosome binding function to retard bacterial multiplication. We hope that this study would form the basis for future experiments (involving *M. tuberculosis* and many other host bacteria) required to unequivocally present a specific or general inhibitory role of DATIN linked to latency or otherwise. Further, mechanistic elucidation of ribosome binding that provides more direct evidence of protein synthesis arrest, would be necessary.
